# Integration of liquid biopsy and pharmacogenomics for precision therapy of EGFR mutant and resistant lung cancers

**DOI:** 10.1186/s12943-022-01534-8

**Published:** 2022-02-24

**Authors:** Jill Kolesar, Spencer Peh, Levin Thomas, Gayathri Baburaj, Nayonika Mukherjee, Raveena Kantamneni, Shirley Lewis, Ananth Pai, Karthik S. Udupa, Naveena Kumar AN, Vivek M. Rangnekar, Mahadev Rao

**Affiliations:** 1 Department of Pharmacy Practice & Science, University of Kentucky, Lexington, KY 40536 USA; 2 Department of Pharmacy Practice, Manipal College of Pharmaceutical Sciences, Manipal Academy of Higher Education, Manipal, Karnataka 576104 India; 3 Department of Radiotherapy and Oncology, Kasturba Medical College, Manipal Comprehensive Cancer Care Centre, Manipal Academy of Higher Education, Manipal, Karnataka 576104 India; 4 Department of Medical Oncology, Kasturba Medical College, Manipal Comprehensive Cancer Care Centre, Manipal Academy of Higher Education, Manipal, Karnataka 576104 India; 5 Department of Surgical Oncology, Kasturba Medical College, Manipal Comprehensive Cancer Care Centre, Manipal Academy of Higher Education, Manipal, Karnataka 576104 India; 6 Markey Cancer Centre and Department of Radiation Medicine, University of Kentucky, Lexington, KY 40536 USA

**Keywords:** EGFR, Liquid biopsy, Lung cancer, Pharmacogenomics, Precision therapy, Tyrosine kinase inhibitors

## Abstract

The advent of molecular profiling has revolutionized the treatment of lung cancer by comprehensively delineating the genomic landscape of the epidermal growth factor receptor (EGFR) gene. Drug resistance caused by EGFR mutations and genetic polymorphisms of drug metabolizing enzymes and transporters impedes effective treatment of EGFR mutant and resistant lung cancer. This review appraises current literature, opportunities, and challenges associated with liquid biopsy and pharmacogenomic (PGx) testing as precision therapy tools in the management of EGFR mutant and resistant lung cancers. Liquid biopsy could play a potential role in selection of precise tyrosine kinase inhibitor (TKI) therapies during different phases of lung cancer treatment. This selection will be based on the driver EGFR mutational status, as well as monitoring the development of potential EGFR mutations arising during or after TKIs treatment, since some of these new mutations may be druggable targets for alternative TKIs. Several studies have identified the utility of liquid biopsy in the identification of EGFR driver and acquired resistance with good sensitivities for various blood-based biomarkers. With a plethora of sequencing technologies and platforms available currently, further evaluations using randomized controlled trials (RCTs) in multicentric, multiethnic and larger patient cohorts could enable optimization of liquid-based assays for the detection of EGFR mutations, and support testing of CYP450 enzymes and drug transporter polymorphisms to guide precise dosing of EGFR TKIs.

**Supplementary Information:**

The online version contains supplementary material available at 10.1186/s12943-022-01534-8.

## Introduction

More than 19.3 million new cases of cancer were reported worldwide in 2020. Globally, lung cancer is the leading cancer that accounts for 11.4% of all cases and is also the leading cause of cancer related deaths [1]. Over the previous 20 years, a focus on genomics research has led to the identification of genomic drivers of lung cancer. The first identified and most broadly studied is epidermal growth factor receptor (EGFR), a transmembrane receptor tyrosine kinase that is part of the ErbB family. EGFR activating mutations act to amplify downstream phosphorylation cascade signaling, resulting in increased cell proliferation and survival. Activating EGFR mutations are the known drivers of lung cancer that accounts for approximately 10 to 15% of non-small cell lung cancer (NSCLC) diagnoses [2–5] and assessment of EGFR mutations is now routinely performed as standard of care.

Tissue biopsy is the gold standard for selecting targeted therapies for NSCLC and current guidelines recommend liquid biopsies to guide initial therapeutic decisions in advanced NSCLC only if obtaining a tissue biopsy is not feasible [6–8]. However, when compared to tissue biopsies, liquid biopsies are less invasive, do not rely on obtaining a tissue biopsy, reduce procedural complications, and importantly can serve as a tool for monitoring EGFR treatment resistance and efficacy. Serial liquid biopsy monitoring throughout a patient’s treatment can allow researchers to identify and understand genomic resistance mechanisms [9–13].

Many liquid biopsies in development are blood-based but testing methods are highly variable. Metastases from a primary tumor requires multiple biological processes that include invasion into the vascular circulation, seeding in distant tissue, and forming a vascular network necessary for cellular survival and proliferation [14]. Blood-based liquid biopsies exploit these tumor characteristics to detect cellular components or genomic contents released by cancer cells into the peripheral blood [14, 15]. Next generation sequencing (NGS) technology has allowed improved detection of cell free DNA (cfDNA) or circulating tumor DNA (ctDNA) and are the only clinically validated methods as companion diagnostics for EGFR mutated NSCLC [16, 17]. Several other blood-based liquid biopsies in development include circulating tumor cells (CTCs), microRNA (miRNA), long non-coding RNA (lncRNA), exosomes, and tumor-educated platelets (TEPs) that have the potential for diagnostics, prognostics, and predicting treatment resistance in EGFR mutated lung cancer [18–26]. Liquid biopsies of pleural effusion fluid are another promising method that is currently being investigated to potentially overcome the limitations of peripheral blood liquid biopsies [27].

**Fig. 1:**
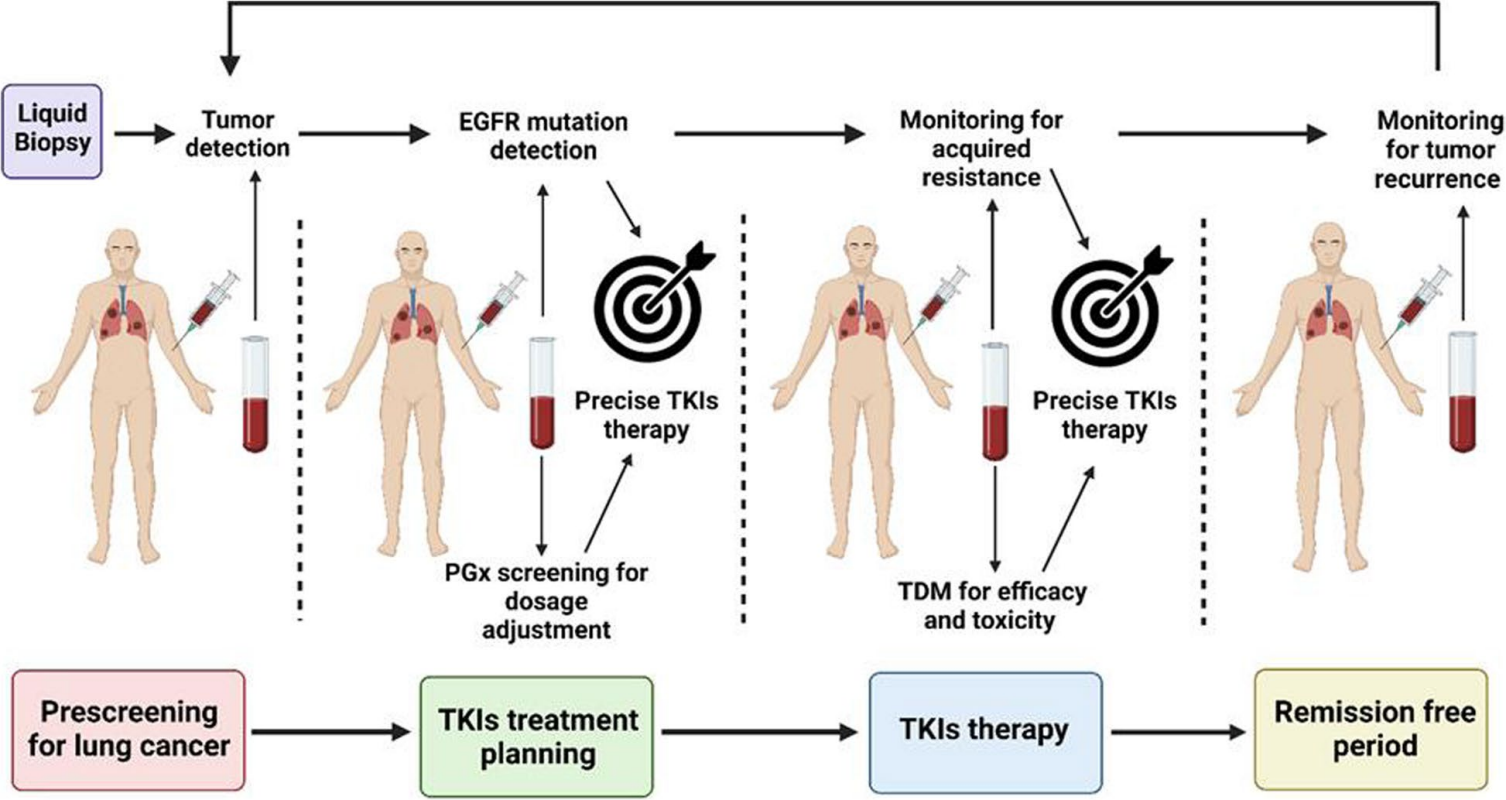
Serial molecular profiling by liquid biopsy and pharmacogenomics across various phases of lung cancer such as screening, treatment planning, monitoring of pharmacotherapy till remission free stage for precision therapy of EGFR mutant and resistant lung cancer. Created with BioRender.com (Agreement number: FS237SI0R8). Abbreviations: EGFR: Epidermal growth factor receptor; TKIs: Tyrosine kinase inhibitors; PGx: Pharmacogenomics; TDM: Therapeutic drug monitoring

An often-overlooked aspect of precision therapy is pharmacogenetic (PGx) variations in drug metabolism. Approximately 80% of drugs available in the United States are metabolized via the cytochrome 450 (CYP450) pathway, including the majority of EGFR TKIs [28, 29]. CYP450 is a family of enzymes involved in oxidation or conjugation of xenobiotics, rendering drugs more hydrophilic and eventually allowing for renal excretion [29, 30]. In addition, multi-drug resistant transporter proteins (MDRPs) such as permeability glycoprotein (P-gp) or breast cancer resistant protein (BCRP) influence xenobiotic transport [31]. P-gp and BCRP are ATP-binding cassette proteins and act as efflux transporters of xenobiotics, including some EGFR inhibitors [32]. Single nucleotide polymorphisms (SNPs) in CYP450s and MDRPs cause variations in pharmacokinetic (PK) and pharmacodynamic (PD) properties of drugs across disease states including cancer [29, 30]. Germline PGx variations in CYP450s and MDRPs can impact the PK/PD properties of EGFR TKIs and subsequently result in increased toxicity or decreased efficacy in certain subsets of the patient population. Pre-emptive testing of germline PGx is not yet standard of care in oncology. With the increasing use of liquid biopsies in clinical practice, there are opportunities to incorporate germline PGx testing given other incidental findings of germline mutations in patients [33]. Such an integrated approach of liquid biopsy and PGx testing could pave the way for precision therapy in lung cancer by tumor detection, dynamic monitoring of EGFR mutations and acquired resistance, as well as aid in the selection of precise drug therapy via serial molecular profiling from blood as shown in Fig. 1.

The purpose of this review is to summarize current literature, opportunities, and challenges for liquid biopsy and PGx testing as precision therapy tools in the management of EGFR mutated lung cancer.

## EGFR TKIs therapy and resistance

The *EGFR* gene encodes a transmembrane protein having 1186 amino acids, with the extracellular region/ectodomain accounting for 621 residues [34]. The *EGFR* gene comprises 28 exons, with the exons 18-21 coding for ATP-binding within the tyrosine kinase domain [35]. Human EGFR is a transmembrane glycoprotein with a glycosylated extracellular domain that binds peptide growth factor ligands, a single transmembrane region, and a cytoplasmic domain with tyrosine kinase activity that plays a key role in the regulation of cellular proliferation, differentiation, survival, and metastasis [36–39]. Actionable driver mutations detected in patients with advanced NSCLC are sensitizing EGFR mutations, which affect about 50% of Asians and 15% of Caucasians [40, 41]. Exon 19 deletions and exon 21 L858R point mutations are the most prevalent EGFR sensitizing mutations, accounting for approximately 90% of mutations in NSCLC and leading to high sensitivity to TKIs [40, 42–46].

Traditionally, platinum-based chemotherapy was the first-line therapy for advanced non-small cell lung cancer (NSCLC). Regardless of clinical characteristics, guidelines recommend that all advanced lung adenocarcinoma patients should be tested for EGFR mutations [47, 48]. Lung adenocarcinoma patients should be assessed for oncogenic drivers and treated with targeted therapy [49] if targetable mutations are present. EGFR TKIs are suggested as the primary therapy for EGFR-mutant patients by the European Society for Medical Oncology (ESMO) [47], American Society of Clinical Oncology (ASCO) [50], and the National Comprehensive Cancer Network (NCCN) [51]. In patients with an EGFR mutation, EGFR TKIs considerably improved clinical outcomes, such as progression-free survival (PFS) and overall response rate (ORR). Patients with lung adenocarcinoma and EGFR mutation have a response rate of up to 81.6% and a PFS of approximately 9.7 to 13.3 months [43]. For metastatic NSCLC patients with EGFR mutations, several phase III clinical trials have found that first-generation and second-generation TKIs are more efficacious than first-line platinum-based chemotherapy [52]. Although most patients with an EGFR mutation are likely to benefit from EGFR TKIs, many develop progressive disease within a year of initiating therapy [53]. Furthermore, long-term efficacy of EGFR TKIs is reduced by acquired resistance.

### History of EGFR TKIs development

The first EGFR inhibitor, gefitinib, was initially approved for the treatment of NSCLC regardless of mutation status based on phase II data, which did not translate to improved outcomes in large phase III trials and led to the withdrawal of gefitinib from the US market unless patients were receiving the drug and benefiting [54]. Around that time, reports emerged regarding the importance of EGFR mutations in predicting response to EGFR inhibitors [55–57]. Subsequent studies of the first-generation EGFR TKIs enrolled patients based on EGFR mutation status and compared outcomes to front line chemotherapy. Gefitinib and erlotinib demonstrated improved PFS in comparison to chemotherapy and were approved as first-line therapy for individuals with EGFR-mutated NSCLC. However, overall survival (OS) was similar, suggesting development of resistance or high rates of cross-over from the chemotherapy arms after trial completion [44, 58–60].

After approvals in the front-line setting, subsequent EGRF inhibitors were compared to gefitinib or erlotinib. Afatinib is another first-generation EGFR TKI which demonstrated a time to treatment failure of 13.7 versus 11.5 months when compared to gefitinib [61]. Osimertinib was assessed in trials that enrolled patients with EGFR L858R (FLAURA), exon 19 deletion (FLAURA), and T790M (AURA3). Osimertinib demonstrated significant OS benefits in the FLAURA trial [62] but not in the AURA3 trial [63]. Despite overcoming EGFR T790M mutation in NSCLC, patients will usually develop other resistance mechanisms, resulting in loss of EGFR TKIs efficacy. Therefore, there is a dire need to understand and monitor treatment resistance mechanisms for further therapy development. Aside from efficacy and resistance, another pertinent aspect of precision oncology for EGFR TKIs is the tolerability of toxicities in different patient populations.

### Mechanisms of acquired resistance to EGFR TKIs

**Fig. 2:**
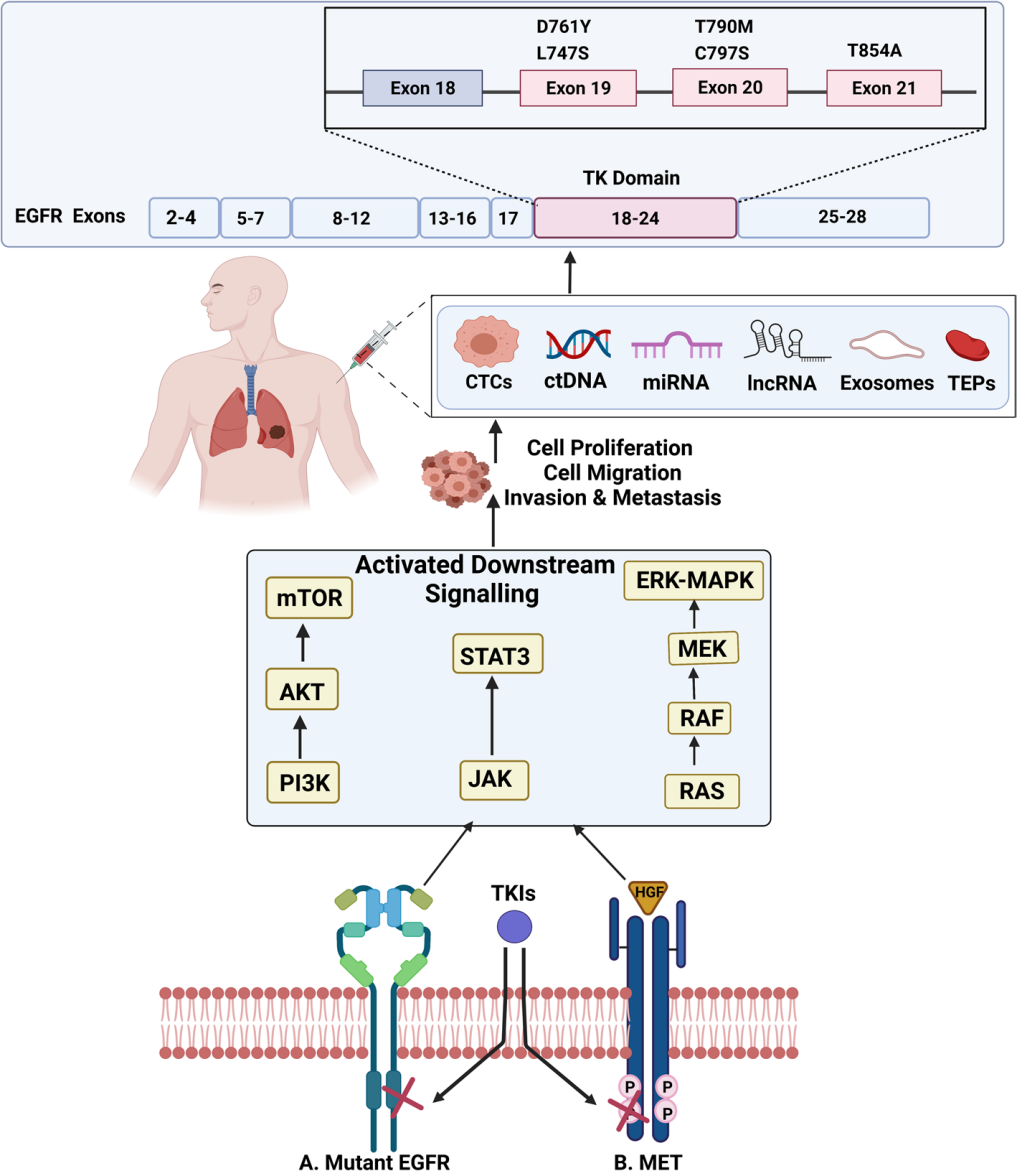
Schematic diagram explaining mechanisms of acquired resistance to EGFR TKIs. **A.** Mutant EGFR confers resistance to binding of TKIs to the tyrosine kinase domain of EGFR. This activates downstream signaling pathways such as the PI3K-AKT pathway, JAK-STAT pathway, RAS pathway and ERK-MAPK pathway. **B.** Overexpression of HGF causes TKI-resistance that activates downstream signaling of the PI3K-AKT pathway, JAK-STAT pathway, RAS pathway and ERK-MAPK pathway. The above pathways result in cell proliferation, cell migration, invasion, and metastasis, that in turn cause the release of CTCs, ctDNA, miRNA, lncRNA, exosomes and TEPs into the bloodstream. Liquid biopsy helps examine these biomarkers and assess the type of mutation. Created with BioRender.com (Agreement number: XF237SHT72). Abbreviations: EGFR: Epidermal growth factor receptor; TK: Tyrosine kinase; CTCs: Circulating tumor cells; ctDNA: Circulating tumor DNA; miRNA: Micro RNA; lncRNA: Long non-coding RNA; TEPs: Tumor educated platelets; mTOR: Mammalian target of rapamycin; AKT: V-akt murine thymoma viral oncogene homolog; PI3K: Phosphoinositide 3-kinase; STAT3: Signal transducer and activator of transcription 3; JAK: Janus Activated Kinase; ERK: Extracellular-signal-regulated kinase; MAPK: Mitogen-activated protein kinase; MEK: Mitogen-activated protein kinase kinase; RAF: Rapidly Accelerated Fibrosarcoma; RAS: Rat sarcoma virus; HGF: Hepatocyte growth factor; TKIs: Tyrosine kinase inhibitors; MET: Mesenchymal epithelial transition factor

Disease progression (based on WHO criteria or RECIST) while on EGFR TKIs is often caused by EGFR resistance to the treatment. It is a major hurdle to overcome in providing the most efficacious treatment to individuals with EGFR-mutant NSCLC [64]. Usually, acquired resistance to EGFR TKIs evolves after a median of 9.2–14.7 months [44, 45, 59, 65]. Target gene modification, alternative pathway activation, and histological or phenotypic transformation are the three prevalent mechanisms of acquired resistance to EGFR TKIs [66] (Fig. 2).

#### EGFR-dependent mechanism: target gene modification

EGFR T790M mutation, which replaces methionine with threonine at position 790 in exon 20 of EGFR, is a common mechanism of resistance to EGFR TKIs. It accounts for 50–60% of the cases [67, 68]. T790M may promote EGFR TKIs such as gefitinib, erlotinib, and afatinib resistance by generating steric hindrance to TKIs binding to the ATP-binding pocket, or by increasing EGFR’s ATP binding affinity. The T790M mutation in EGFR may restore the mutant receptor’s affinity for ATP, lowering the effectiveness of competitive inhibitors [67, 69]. Detection of EGFR T790M status is important since it affects treatment choice indicating the use of the third-generation EGFR TKI, osimertinib as a second-line therapy [70].

#### EGFR-independent mechanism: alternative pathway activation

The activation of alternative or bypass pathways can also produce acquired resistance. The most prevalent bypass mechanism is MET amplification, accounting for 5–10% of patients with resistance to TKIs [66, 71, 72]. The MET gene encodes the receptor tyrosine kinase c-MET, and binding of MET to its ligand, hepatocyte growth factor (HGF), causes tyrosine phosphorylation of the receptor and initiation of downstream signaling pathways, such as phosphoinositide 3-kinase (PI3K) and V-akt murine thymoma viral oncogene homolog (AKT), signal transducer and activator of transcription 3 (STAT3), rat sarcoma virus (RAS), and mitogen-activated protein kinase (MAPK). Combination trials of MET directed therapies and T790M targeting inhibitors are important because MET amplification and T790M mutation are not mutually exclusive [73]. MET gene amplification can initiate PI3K-AKT pathway independently of EGFR by ERBB3 dimerization and signaling [72]. However, the MET amplification threshold that confers acquired resistance to TKI-therapy is yet to be determined. EGFR TKIs resistance is also promoted by overexpression of HGF, the ligand of MET oncoprotein [74]. Other alternative mechanisms that have been reported to cause resistance to TKIs, including KRAS mutation, BRAF mutation [75, 76], HER2 amplification [77], phosphatidylinositol 4,5-bisphosphate 3-kinase catalytic subunit alpha isoform (PIK3CA) mutation [78], and enhanced expression of the receptor tyrosine kinase AXL [79].

#### Histological and phenotypic transformation

During or after treatment with EGFR TKIs, a subset of individuals (i.e., 5–10%) with NSCLC and EGFR mutations develops histologic transformation of adenocarcinoma into small-cell lung cancer (SCLC) [78, 80–82].

Prolonged EGFR inhibition in NSCLC can result in the appearance of SCLC-like histologic, genetic, and pharmacological sensitivity profiles. The epithelial-to-mesenchymal transition (EMT) program has also been linked to SCLC transformation, as demonstrated by decreased expression of E-cadherin and enhanced expression of vimentin [78]. EMT was reported as a mechanism of resistance to TKIs, with EMT identified in 2 of 37 (i.e., 5%) patients in tumor specimens obtained after EGFR treatment and SCLC transformation [78]. Slug, ZEB1, Snail, and AXL are examples of EMT transcription factors that undergo alterations due to acquired resistance to TKIs [83, 84].

### Liquid biopsy in EGFR mutant NSCLC detection

#### Comparative superiority over tissue biopsy-studies with results in EGFR mutant/resistant lung cancer

Liquid biopsy efficiently analyzes CTCs, ctDNA, miRNA, lncRNA, exosomes, and TEPs [23, 25, 26, 85]. Table 1 summarizes the characteristics of the biomarkers with their corresponding isolation techniques, clinical applications, and limitations. ctDNAs formed by the DNA fragments are released into the bloodstream by cell death, especially via necrosis [103, 104]. ctDNA can be extracted from a variety of bodily fluids, including saliva, sputum, CSF, urine, and pleural secretions, in addition to plasma [105, 106]. Despite its moderate sensitivity, urine ctDNA is a viable alternative for detecting EGFR mutations [107]. With a concordance of 84.62% among all patients, [108] tissue biopsy and liquid biopsy using ctDNA can have distinct effects on the prognosis and treatment strategies of EGFR mutant non-small cell lung adenocarcinoma. Although ctDNA is approved for the detection of EGFR mutant in NSCLC patients, [109] adenocarcinomas are more likely to be detected by the established miRNA markers over squamous cell carcinoma [110]. A retrospective study of 308 lung cancer patients who had re-biopsy and 118 patients who had liquid biopsy, found that 134 patients (43.5%) in the re-biopsy group and 49 patients (41.5%) in the liquid biopsy group tested positive for EGFR T790M. The liquid biopsy’s specificity and sensitivity for detecting T790M was 84.4 and 34%, respectively. The study showed that 75.0% of the patients in the liquid biopsy group and 52.3% of the patients in the re-biopsy group, who tested positive for T790M mutation were likely to get treated by a third-generation TKI [10]. Sacher et al. prospectively assessed 180 patients to determine the correlation between tissue re-biopsy and liquid biopsy. The sensitivity, specificity, and positive predictive value of ctDNA based T790M detection using digital droplet polymerase chain reaction (ddPCR) was reported to be 77.1, 63.2, and 79%, respectively [111].

**Table 1:** The characteristics of the biomarkers with their corresponding isolation techniques, clinical applications and limitations

Biomarkers	Isolation technique	Clinical application	Limitations	References
Circulating tumor cells (CTCs)	Immunomagnetic enrichment	▪ Prognosis ▪ Treatment	▪ Reproducibility ▪ Sensitivity ▪ CTC without epithelial marker could not be detected ▪ Difficult to use with whole blood ▪ Low purity of detected CTC ▪ Cannot process whole blood ▪ High detection cost	[86]
	Immunomagnetic isolation	▪ Diagnosis ▪ Prognosis	▪ Reliance on EpCAM and CK ▪ Variation of expression of EpCAM and CK across cancers ▪ Lack of selectivity ▪ High detection cost	[87]
	Magnetic beads	▪ Prognosis ▪ Treatment	▪ Increased contamination with WBC ▪ Requires more blood	[88]
	Microfluidic positive immunocapture (CTC-chip)	▪ Prognosis ▪ Diagnosis	▪ Shear force might affect cell viability and attachment ▪ Slow rate processing ▪ Limited volume	[89]
	Size based separation (filter-based isolation)	▪ Prognosis ▪ Treatment	▪ Prone to clogging ▪ Requires high volume of blood ▪ Sample may be adulterated	[90]
	Density gradient separation	▪ Prognosis ▪ Treatment	▪ Loss of large CTC and cell aggregates ▪ Low purity	[91]
	Inertial focusing	▪ Prognosis ▪ Diagnosis ▪ Treatment	▪ Morphological deformation of the captured cell	[92]
	Single cell sequencing	▪ Prognosis ▪ Treatment	▪ Poor reproducibility ▪ False positives and false negatives ▪ Allele deletion ▪ Sequencing errors	[93]
Circulating tumor DNA (ctDNA)	Manual (DNA purification)	▪ Detection ▪ Prognosis ▪ Treatment initiation and monitoring	▪ Low accuracy and precision	[94]
	Automated (ccfDNA purification)		▪ Requires adequate control for downstream application ▪ Only for use with plasma prepared from human whole blood samples collected in EDTA tube ▪ Not for use in diagnostic procedures	
ctRNA (miRNA, lncRNA)	Northern blot analysis	▪ Diagnosis	▪ mRNA degradation during electrophoresis ▪ Low sensitivity ▪ Detection with multiple probes is difficult	[95]
	Microarray	▪ Diagnosis ▪ Therapeutic response prediction	▪ Standardization and optimization ▪ Low specificity ▪ Low reproducibility ▪ High cost of a single experiment ▪ Unsuitable for clinical experiences	[96]
	RT-qPCR	▪ Diagnosis ▪ Treatment initiation and monitoring	▪ Amplification bias ▪ No template controls ▪ Cannot perform multiple detection	[97]
	Liquid chip technology	▪ Diagnosis ▪ Prognosis ▪ Treatment ▪ Resistance monitoring	▪ In vivo validation ▪ Difficult to scale up	[88]
Exosomes	Ultracentrifugation-based	▪ Early diagnosis ▪ Prognosis	▪ Contamination and exosome loss ▪ Low recovery ▪ Laborious	[98]
	Size-based	▪ Diagnosis	▪ Deformation of EVs ▪ High risk of chip clogging ▪ Long run time	[99]
	Immunoaffinity capture-based	▪ Diagnosis	▪ Antibody cross reactivity ▪ Possible detection of non-EV particles ▪ Only exosomes with targeted proteins can be separated ▪ Low yield ▪ Tumor heterogeneity hinders immune recognition ▪ Time consuming ▪ Expensive	[98]
	Microfluidics based	▪ Diagnosis	▪ Lack of standardization and method validation ▪ Moderate to low sample capacity	[100]
Tumor-educated platelets (TEPs)	Spliced TEP mRNA	▪ Diagnosis ▪ Treatment monitoring	▪ Complex isolation technique ▪ Fragility of TEPs	[101, 102]

*Abbreviations*: *CTCs* Circulating Tumor Cells, *EpCAM* Epithelial cell adhesion molecule, *CK* Cytokeratin, *WBC* White blood cell, *ctDNA* Circulating tumor DNA, *ccfDNA* Circulating cell free DNA, *EDTA* Ethylenediamine tetraacetic acid, *ctRNA* Circulating tumor RNA, *miRNA* MicroRNA, *lncRNA* Long non-coding RNA, *mRNA* Messenger RNA, *RT-qPCR* Quantitative real-time polymerase chain reaction, *EVs* Extracellular vesicles, *TEPs* Tumor-educated platelets

In liquid biopsy, the plasma samples are predominantly analyzed using quantitative polymerase chain reaction (qPCR), digital PCR (dPCR) or ddPCR, and NGS [112]. Feng Li et al. compared the concordance of electric field-induced release and measurement (EFIRM)-based liquid biopsy with ddPCR to establish the superiority of the former. The study concluded a 100% sensitivity for EFIRM as opposed to 84.6% sensitivity in the detection of EGFR mutation in plasma samples. This is because of the limitation of PCR to amplify short DNA fragments (shorter than 70 bps) [113]. The largest prospective, multicenter trial on cfDNA conducted by Leighl et al. concluded that cfDNA testing could identify the biomarkers with a sensitivity and specificity, comparable to tissue genotyping [114]. Use of cfDNA yielded faster results and increased the biomarker discovery rate, although the median turnaround time (TAT) recorded in this study for the first 10 patients was 14 days (range, 11-30 days) and 7 days (range, 5-9 days) for the last 10 patients [114]. Often false negative results are obtained due to varied tumor localization and volume, irregular cfDNA shedding with tumor evolution [115] or patients receiving treatment [116]. A diagnostic tool comprising of a panel of miR-21, miR-126, miR-210, miR-486-5p detected NSCLC with a sensitivity and specificity of 86.2 and 96.5% respectively [110]. The SensiScreen® EGFR Liquid kit that was commercially launched recently detects EGFR mutations (T790M, L858R, exon 19 deletions) at a higher sensitivity and specificity, which outperforms the established assay platforms with a robust ability to detect single copy mutations [117].

LncRNAs could serve as potential predictive and prognosis markers for EGFR resistant and mutant lung cancers as, they have been implicated in the regulation of chemosensitivity, radiosensitivity, and sensitivity of EGFR targeted therapies in lung cancers through diverse mechanisms [25]. LncRNA LINC00460 overexpression in EGFR-mutant lung adenocarcinoma was reported to be associated with poorer response to EGFR TKI therapies. Drug molecules that could target knockdown or knockout of LINC00460 may represent potential therapeutic strategy for overcoming EGFR TKIs resistance and consequently improve the prognosis of EGFR mutant lung cancer patients [118]. LncRNA bladder cancer associated transcript 1 (BLACAT1) knockdown was reported to reverse afatinib resistance in NSCLC through modulation of STAT3 signalling [119]. Several other lnRNAs such as BC087858, metastasis associated lung adenocarcinoma transcript 1 (MALAT-1) have been implicated in the promotion of EGFR TKIs resistance in lung cancer via regulation of EMT process [120]. T790M mutation detection by simultaneously capturing and interrogating exosomal RNA/DNA and cfDNA (exoNA) had 92% sensitivity and 89% specificity using results of tumor biopsy as gold standard [26]. Another qPCR- based test that assessed mutations within EGFR using exoNA of NSCLC patients reported an overall sensitivity of 90% for L858R, 83% for T790M and 73% for exon 19 indels with specificities of 100, 100, and 96% respectively [121]. Combined approach of using exosomal RNA and ctDNA among EGFR mutant NSCLC patients improved sensitivity of EGFR mutation detection [122]. Using short length exosomal DNA and RNA (exoTNA) of 200 bp length could potentially serve to be a sensitive biomarker for detection of EGFR mutants in NSCLC patients having low copy numbers of target mutation [123]. Several TEP biomarkers that could potentially be utilized for early screening of NSCLC have been reported [23]. Early stage and late-stage NSCLC were detected with an accuracy of 81 and 88% respectively from TEP RNA biomarker panel [124]. EGFR mutation detection in NSCLC patients was observed with 87% accuracy using the TEP-derived RNA analysis [125].

#### Utility in detecting acquired resistance to EGFR TKIs

EGFR T790M mutation was reported to be the primary acquired resistance to TKI therapy, followed by MET amplification, HER2 amplification and epithelial to mesenchymal transition [126, 127]. The AURA2 trial investigated the efficacy of osimeritinib, an irreversible tyrosine kinase inhibitor, in treating patients with advanced NSCLC and either EGFR-TKI sensitizing or EGFR T790M mutations. The trial demonstrated a median PFS of 9.9 months and osimertinib was well tolerated among the treated patients [128]. Consistently, the AURA Phase II extension study demonstrated a median PFS of 12.3 months with a tolerable safety profile [129]. Based on these findings, osimeritinib is now regarded in the first line of treatment in patients with EGFR T790M mutant NSCLC. Over the course of treatment with first or second-generation TKIs, patients usually acquire several mutations, including EGFR, BRAFV600E, and ERBB2 mutations; ALK, ROS1, NTRK, and RET fusion; MET amplification and MET exon 14 skipping variants that can be assessed to identify patients for subsequent targeted therapy [114].

Until recently, the only way to identify T790M status was by re-biopsy of tumor tissue. However, liquid biopsy genotyping has recently become a more appealing option to tissue re-biopsy, particularly for detecting the growing number of resistance mutations that may develop during therapy [71, 78]. Several studies have addressed the usefulness of liquid biopsy in detecting molecular alterations that cause resistance mechanisms [130, 131]. The first report of ctDNA study with T790M in plasma was published in 2009 [132]. The identification of T790M by whole-exome sequencing of ctDNA using longitudinal blood-based EGFR testing was initially reported in 2013 [133]. Utilizing both ctDNA and CTCs, many studies have demonstrated the value of using liquid biopsies to detect EGFR resistance mutations [134–141]. Based on this finding, the NCCN and the ESMO guidelines both suggest plasma genotyping as an alternative to tissue-based testing, although secondary re-biopsy is recommended to confirm a negative plasma evaluation of T790M [6, 142]. Resistance of T790M mutants to EGFR TKIs was studied by analyzing ctDNA using CAPP-Seq in patients treated with rociletinib [141]. Chabon et al. identified a shorter PFS accompanied with novel resistance mechanism (activating KRAS, EGFR L798I) upon treatment with a third-generation TKI [141]. A study conducted by Rachiglio et al. investigated the role of concomitant driver mutations (MET, ERBB2, NRAS, BRAF, KRAS, PIK3CA) on the outcome of 133 NSCLC patients who received TKIs. Patients with concomitant driver mutations had a significantly lower PFS than those with only an EGFR mutation (7 vs. 11.3 months; *p* = 0.04) implying that a subset of EGFR mutant tumors have concomitant driver mutations, that could affect the efficacy of first-generation EGFR TKIs [143]. Another cfDNA analysis reported that after progression on EGFR TKIs, 48.5% of plasma samples were positive for KRAS mutation, with 39.4% of those having a KRAS and EGFR co-mutation [144]. Though SCLC transformation is difficult to detect with liquid biopsy, a recent study showed that ctDNA can be examined in terms of changes in global copy number to track its dynamics in patients with SCLC transformation [145]. The mechanism of SCLC transformation is still largely undefined. However, it is possible that deletion of the retinoblastoma gene (RB) plays a role [146]. Completed and ongoing trials on liquid biopsy for the detection of EGFR mutant NSCLC are tabulated in the Supplementary Table 1 [147].

#### Utility in detecting prognosis

Tumor mutational burden (TMB), a surrogate for overall neo-antigen load [148] can be analyzed using tissue and blood-based assays. The CheckMate-026 trial reported an association between high tissue TMB (tTMB) and the clinical efficacy of nivolumab in NSCLC. An independent association between blood TMB (bTMB) and PFS prediction in patients receiving atezolizumab monotherapy was reported in NSCLC patients. The study also exemplified a high concordance between bTMB and tTMB when run on the same ctDNA sample [149]. A liquid biopsy with NGS can help detect tumor progression and accompanying multiple genetic alterations [150]. The trial, Tracking Non–small-Cell Lung Cancer Evolution Through Therapy (TRACERx) by Hanjani et al. analyzed chromosomal instability and genome doubling prospectively by whole exome sequencing (WES) to assess the driver events in NSCLC to predict a poor prognosis. Altered genomic co-occurrence with tumor progression was inferred to influence the patient’s response to TKIs [151]. A group of advanced EGFR mutant patients were assessed for multiple co-occurring genetic alterations. cfDNA was used to identify the co-occurrence of the genetic alterations within the WNT/CTNNB1, BRAF, MET, PIK3CA, MYC, and the cell cycle pathways (CDKN2A loss and CDK6 CNG). Analysis of longitudinal tumor biopsy based whole exome sequencing and cfDNA was consistent with the genomic alterations [152]. Genomic profiling of ctDNA samples can identify therapeutic targets by locating driver and resistance mutations Analysis of the ctDNA of 8388 advanced lung adenocarcinoma and NSCLC patients by 70 gene NGS panel (Guardant360 assay) identified oncogene driver mutations in 48.8% of the samples, the most frequent mutations being EGFR followed by KRAS. The study subsequently recorded a 65% increase in biomarker detection over tissue, where one half of the patients received targeted therapy [153]. Oxnard et al. proposed plasma genotyping of cfDNA as a screening method for T790M preceding EGFR resistance biopsy. However, with a 30% false negative rate of plasma genotyping, tissue genotyping is still required for some patients. Therefore, the concomitant use of tissue and plasma genotyping is the new paradigm in determining T790M resistance management [154].

## Pharmacogenomics of TKIs and implications pertaining to PK/PD responses

### Metabolism of EGFR TKIs

EGFR TKIs are small molecules that are highly protein bound and metabolized via the CYP450 system. The majority are also substrates of P-gp and BCRP [155]. P-gp and BCRP are a family of ATP-binding cassette (ABC) transporters and are encoded by the genes ABCB1 and ABCG2, respectively [156, 157]. A summary of EGFR TKI metabolism is listed in Table 2. Gefitinib is a first-generation EGFR TKI primarily metabolized by CYP3A4, CYP2D6, and to a minor extent, CYP3A5 [158–163]. CYP1A1 may be involved in gefitinib metabolism but PK implications have yet to be characterized since CYP1A1 is typically expressed in extrahepatic locations such as the lungs [164]. Gefitinib is also known substrate of P-gp and BCRP [165–167]. Erlotinib is primarily metabolized by CYP1A2 and 3A4 but also induces 3A4 expression to a minor extent [163, 168]. P-gp and BCRP may also contribute to erlotitnib clearance [169]. Afatinib is the only EGFR TKI that is not metabolized via the CYP450 system due to its strong covalent binding to plasma proteins and is primarily excreted through the feces [170, 171]. Afatinib is both a substrate and inhibitor of P-gp and BCRP [170, 172, 173]. Dacomitinib is extensively metabolized by CYP2D6 into its active metabolite, contributing to the long half-life of the drug [174]. Dacomitinib’s clearance is neither known to be impacted by P-gp nor by BCRP [175]. Osimertinib is primarily metabolized by CYP3A4, and is minimally cleared by P-gp and BCRP [176, 177]. Mobocertinib is the newest oral EGFR TKI that received accelerated approval in September 2021 by the FDA for EGFR exon 20 deletion NSCLC. Mobocertinib is metabolized by CYP3A4 and 3A5 to form two active metabolites [178]. It is unknown if mobocertinib is a P-gp or BCRP substrate at this point.

**Table 2:** A summary of metabolism of EGFR TKIs drugs

EGFR TKIs	Generation	Metabolism	Drug-drug interactions	MDRP substrates
Gefitinib	First	CYP3A4, CYP2D6, CYP3A5 (minor)	▪ CYP3A4, CYP2D6 inhibitors may increase serum concentration ▪ CYP3A4, CYP2D6 inducers may decrease serum concentration	P-gp and BCRP
Erlotinib	First	CYP1A2, CYP3A4	▪ CYP3A4, CYP2A1 inhibitors may increase serum concentration ▪ CYP3A4, CYP2A1 inducers may decrease serum concentration ▪ Erlotinib reduce serum concentrations of other CYP3A4 substrates	P-gp and BCRP
Afatinib	First	None	▪ P-gp inhibitors may increase serum concentration ▪ P-gp inducers may decrease serum concentration	P-gp and BCRP
Dacomitinib	Second	CYP2D6	▪ CYP2D6 inhibitors may increase serum concentration	None
Osimertinib	Third	CYP3A4	▪ CYP3A4 inhibitors may increase serum concentration ▪ CYP3A4 inducers may decrease serum concentration ▪ Osimertinib may increase serum concentrations of other P-gp/BCRP substrates	P-gp and BCRP
Mobocertinib	Third?	CYP3A4, CYP3A5	▪ CYP3A4/5 inhibitors may increase serum concentration ▪ CYP3A4/5 inducers may decrease serum concentration	Unknown

*Abbreviations*: *TKIs* Tyrosine kinase inhibitors, *MDRP* Multi-drug resistant transporter protein, *P-gp* Permeability glycoprotein, *BCRP* Breast cancer resistant protein

### Single nucleotide polymorphisms, allele frequencies and phenotypes

Single nucleotide polymorphisms (SNPs) occur when a single DNA base differs between individuals and varies across race and ethnic groups [179]. SNPs are functionally categorized by phenotypic impact. In general, normal metabolizers (NM) are present in majority of the population and have wild type functional enzyme activity. Ultra-rapid metabolizers (UM) have increased enzyme activity compared to normal metabolizers. Poor-metabolizers (PM) have limited to no enzyme activity. Intermediate metabolizers (IM) have enzyme activities between PM and NMs [180]. The phenotypic impact of SNPs in drug metabolizing enzymes can vary, ranging from benign to a significant loss or gain of enzyme activity and phenotype reporting based on ethnicity, clinical guidelines, and laboratories was inconsistent in previously published literature [181].

CYP2D6 accounts for 25% of all drug metabolism and has the most polymorphic variability in the CYP450 family [182]. In 2019, consensus guidelines recommended standardization of CYP2D6 phenotype definitions based on an activity score [183, 184]. Notably, inconsistencies in phenotype assignments may occur due to changes to phenotype definition as more evidence become available [182, 185]. Although CYP3A4 is the major metabolizer of drugs and its polymorphic variability has been extensively studied, there is little evidence supporting a role for CYP3A4 polymorphisms in changing the metabolism of substrates. One possible explanation could be the structural similarities between subfamilies, leading to erroneous identification of CYP3A4 [186, 187]. The CYP1A2 -163C > A SNP polymorphism (haplotype CYP1A2*1F) has increased enzyme activity for substrates such as caffeine and is the most well characterized CYP1A2 polymorphism [188]. However, at present, there is a lack of evidence for assigning CYP1A2 phenotypes due to relative infancy CYP1A2 polymorphism research. Similarly, SNP polymorphisms of ABCB1 and ABCG2 have been reported, however but currently, standardized definitions for phenotypes are lacking.

### Smoking, CYP1A2, polymorphisms and erlotinib

Cigarette smoke produces polycyclic aromatic hydrocarbons (PAH), a class of compounds known to induce the expression of CYP1A2 [189, 190]. Several hypotheses point towards a transcriptional mechanism for CYP1A2 induction by PAH. PAH may act as a ligand for arylhydrocarbon receptor, which is an intracellular receptor involved in downstream signaling of CYP1A2 transcription [191, 192]. Another explanation may be epigenetic changes via chromatin remodeling and reduced expression of histone deacetylase 2 resulting in increased transcription of CYP1A2 [193, 194]. Induction of CYP1A2 expression increases clearance of erlotinib leading to reduced plasma exposure and subsequently lowering efficacy [195]. Even though EGFR mutations occur more frequently in non-smokers with lung cancer, managing smokers and former smokers on erlotinib treatment remains challenging clinically. Smokers and former smokers had a 3.9% response rate compared to 24.7% in a selected subset of population on erlotinib [196]. The higher number of former and current smokers may also have contributed to minimal OS benefit in the overall population. Later erlotinib trials that enrolled patients based on EGFR mutation status demonstrated significantly improved efficacy of erlotinib. However, smokers or former smokers still made up approximately 30% of the study population [58, 126, 197] that may likely reflect the proportion of smokers or former smokers with EGFR mutated lung cancer in the real world. A pharmacokinetic model demonstrated a decrease in erlotinib exposure by more than 20% in patients exposed to cigarette smoking [198]. Another study has also showed that doubling erlotinib dose from 150 mg to 300 mg in current smokers resulted in similar plasma concentrations of erlotinib compared to non-smokers on 150 mg dose, suggesting a potential need for higher doses of erlotinib to achieve adequate efficacy [199]. Based on the study by Hughes et al. up to 300 mg once daily of erlotinib is recommended for current smokers.

Despite studies suggesting the detrimental impact of smoking on erlotinib exposure and efficacy, there are still varying extents of CYP1A2 induction by cigarette smoking that could be due to other epigenetic factors affecting CYP1A2 expression [200–203]. Polymorphisms in the NR1I3 gene which codes for the constitutive androstane receptor is known to upregulate the transcription of CYP1A2 [200, 204]. Constitutive androstane receptor is also known to interact with the PAH pathway of CYP1A2 induction. Another epigenetic factor may be due to the influence of methylation on CYP1A2 expression in hepatocytes [205]. Previous literature also suggests that certain CYP1A2 polymorphisms such as CYP1A2*1F may be induced to a larger extent in smokers [202, 203]. Contributions to erlotinib metabolism by CYP3A4, P-gp, and BCRP, may also explain the variation in CYP1A2 induction by cigarette smoke. Therefore, there is currently limited recommendation for phenotype definition and lack of guidelines for CYP1A2 based PGx testing with erlotinib use.

### Polymorphisms in CYP3A4

Phenotyping studies assessing the effect of CYP3A4 variants on erlotinib metabolism were inconclusive and pre-emptive testing is not currently recommended. A PK study in a Korean population showed no difference in AUC exposure and C_max_ of erlotinib in patients with CYP3A4 polymorphisms [206]. In a similar study, a polymorphism in CYP1A2*1 M resulted in a higher C_max_. One explanation for the lack of evidence for supporting PGx guided dosing for erlotinib may be due to multiple metabolic pathways involved in erlotinib clearance. Another contributing factor could be auto-induction of CYP3A4 by erlotinib. CYP450 induction often occurs on a transcriptional level and takes up to 2 weeks for increased expression, which may be missed in studies that do not assess steady state erlotinib levels [206, 207]. There are limited studies evaluating the impact of CYP3A4 SNPs and gefitinib, osimertinib, and mobocertinib metabolism. Furthermore, there is a lack of evidence suggesting that CYP3A4 polymorphisms alter PK/PD parameters of EGFR TKIs. Similar to CYP1A2, phenotypes for CYP3A4 are yet to be defined and there is lack of guidance supporting testing for CYP3A4 polymorphisms to guide dosing of EGFR TKIs.

### Polymorphisms in CYP2D6

Numerous studies have evaluated associations of gefitinib adverse effects, such as rash and hepatotoxicity, in patients who have reduced CYP2D6 metabolizing phenotypes. A study enrolled Japanese patients who developed transaminitis after starting gefitinib, but did not find significant differences in CYP2D6 polymorphisms [208, 209]. Similarly, another study assessing Japanese patients who were extensive metabolizers (EM) and IMs of CYP2D6 found higher active metabolite concentrations, but higher concentrations were not associated with increased adverse effects [209]. In a third study in Japanese patients, Suzumura et al. reported patients with CYP2D6 *10/*10 polymorphism, defined as a reduced activity phenotype, had an increased risk of rash with gefitinib compared to patients on erlotinib [210]. The conflicting evidence supporting a relationship between increased gefitinib adverse effects and CYP2D6 polymorphisms are multifactorial. This may partly be explained by the fact that previous studies utilized the EM or rapid metabolizer phenotype of CYP2D6, which has been removed and re-classified under NM [183]. The lack of standardized CYP2D6 definitions that may also have contributed to differences in testing and selection of alleles of interest remains a challenge today [211]. A recent study of dacomitinib in Chinese patients with IM and EM CYP2D6 polymorphisms found insignificant changes to exposure of primary metabolite of dacomitinib between the two groups [212]. However, it was noted by the authors that CYP2D6 EM in the Chinese population had a 53.5% metabolite to parent exposure ratio compared to 25.4% in a Western CYP2D6 EM population, suggesting that polymorphism and PGx testing may be impacted by ethnicity.

### Polymorphisms in ABCB1 and ABCG2

P-gp and BCRP are primarily expressed along the luminal intestinal wall and blood brain barrier and prevent diffusion of xenobiotics across membranes into the blood circulation and central nervous system, respectively [31]. Polymorphisms in ABCB1 and ABCG2, have been shown to correlate with expression of P-gp and BCRP, respectively. ABCB1 and ABCG2 polymorphisms resulting in increased expression of P-gp and BCRP may reduce bioavailability of a substrates like gefitinib, erlotinib, afatinib and osimertinib resulting in lower systemic exposure [213, 214]. On the other hand, reduced expression of P-gp and BCRP has been purported to increase bioavailability resulting in increased toxicities [167, 215]. Endo-Tsukude et al. reported marginal increase in rash among Japanese patients harboring ABCB1 1236C > T genotypes, however the differences were not significant [216]. A study led by Fukudo and colleagues found that Japanese patients harbouring ABCG2 421C > A SNP polymorphism had increased plasma exposure of erlotinib which is associated with increased diarrhea [217]. However, another study in Japanese patients by Akasaka et al. did not find an increased risk of diarrhea in patients with ABCG2 421C > A polymorphism [218].

An increase in P-gp and BCRP activity or expression along the luminal membrane of the blood brain barrier may decrease central nervous system (CNS) penetration of EGFR TKIs. In vitro and preclinical models of gefitinib, [165, 166] erlotinib, [219–221] and osimertinib [177] have suggested that less CNS penetration was achieved in patients with increased expression of P-gp and BCRP. A decrease in CNS penetration can have detrimental impacts on patients with CNS metastases, which confers poorer prognosis. Alternatively, a decreased expression of P-gp and BCRP may theoretically increase CNS toxicity although there is no evidence published yet.

In addition to germline PGx differences in P-gp and BCRP, cancer cells have been shown to upregulate expression of these transporters and prevent chemotherapy from reaching their intracellular target tissue. Increased P-gp and BCRP contributes to chemoresistance and subsequent treatment failure.

**Fig. 3:**
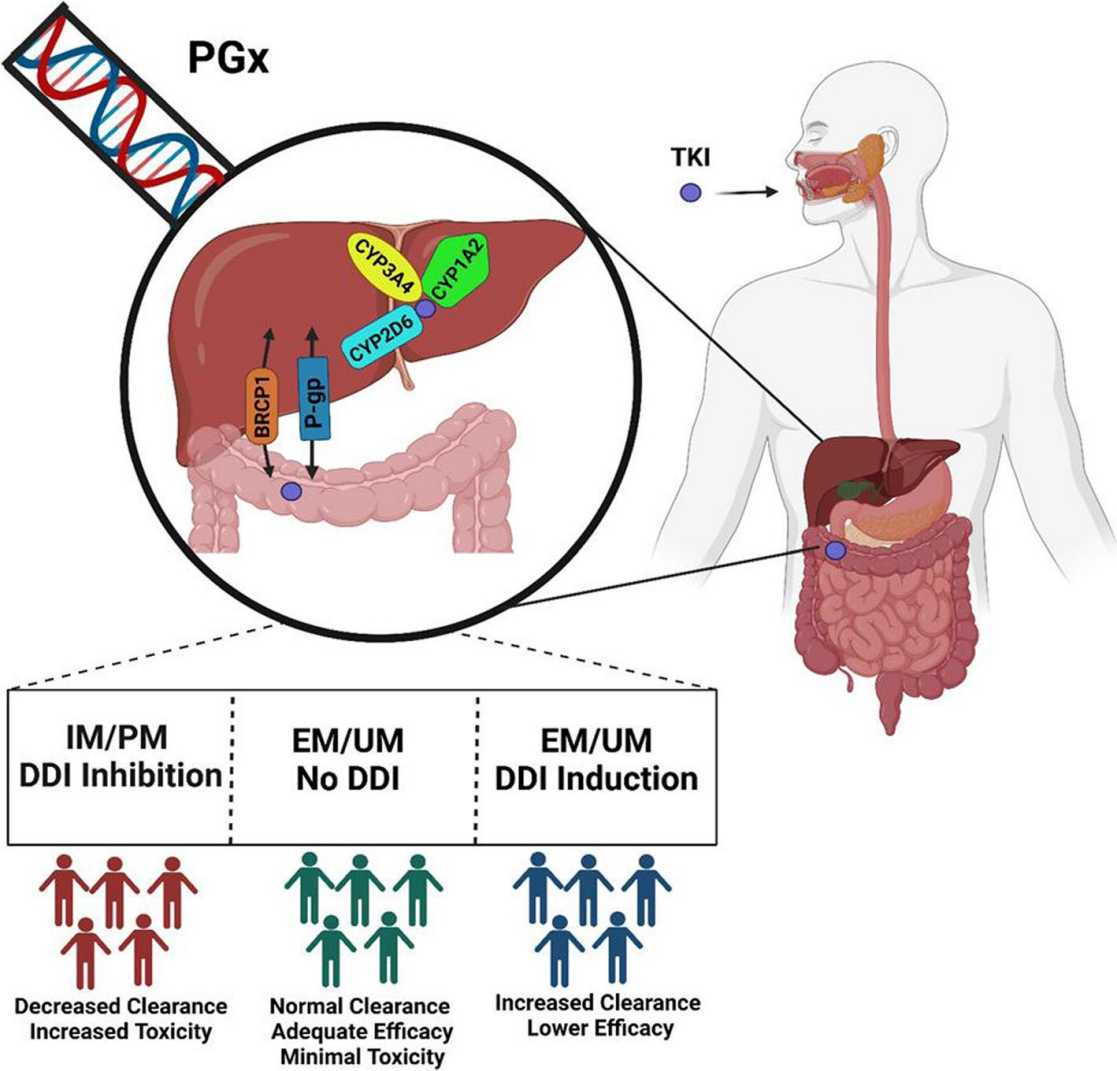
Pharmacogenomic screening of CYP450 enzymes and drug transporters for TKIs could help in stratifying the population into various categories of drug responders. Created with BioRender.com (Agreement number: II237SHPD4). Abbreviations: PGx: Pharmacogenomics; TKI: Tyrosine kinase inhibitors; BCRP: Breast cancer resistant protein; P-gp: Permeability glycoprotein; IM: Intermediate metabolizers; PM: Poor metabolizers; EM: Extensive metabolizers; UM: Ultra rapid metabolizers; DDI: Drug-drug interaction

Current evidence does not support a role for pharmacogenetic dose adjustment of EGFR TKIs. Available studies are usually small, with variable methodologies and conflicting results. Larger studies that comprehensively evaluate the impact of polymorphisms on drug exposure and outcome are needed to optimize precision dosing as shown in Fig. 3.

### Alternative strategies and future directions

It is generally accepted that plasma concentrations are associated with drug effects, both efficacy and toxicity. While SNPs contribute to drug plasma concentrations, a variety of other factors, including drug-drug interactions, food effects, and body size are other contributing factors. By directly measuring drug concentrations, and adjusting dose based on concentration, therapeutic dose monitoring (TDM) can potentially overcome factors that limit the utility of PGx testing. Many analytical methods are reported for erlotinib, [222–225] gefitinib [225–229] and osimertinib [230, 231]. Despite the abundance of methods for detecting EGFR TKI plasma concentrations, TDM of EGFR TKIs are not yet clinically validated or implemented routinely in practice. Identification of a target concentration associated with activity, as well as clinical trials demonstrating that TDM outperforms routine clinical care is required prior to clinical implementation.

Cancer cells are known to develop chemoresistance through upregulation of ABCB1 and ABCG2 expression, preventing chemotherapy from reaching the target tissue. Gefitinib, erlotinib, afatinib, and osimertinib, are substrates of P-gp and BCRP, and a potential strategy is to combine these agents with P-gp inhibitors, thereby re-sensitizing tumors to chemotherapy. Ongoing studies are required to develop these potentially effective combinations. Liquid biopsies can complement such synergistic strategies in clinical practice by testing for upregulation in P-gp and BCRP expression throughout a patient’s treatment.

Traditionally, early phase dose finding clinical trials rely upon a “3 + 3” maximum tolerated dose design. The paradigm shift toward targeted therapy has called for different approaches for optimizing targeted therapy dosing [232]. Preclinical models of targeted therapies utilize receptor saturation or inhibition of phosphorylation activities during the drug development process. It has been shown that EGFR saturation in preclinical models corresponds to anti-tumor efficacy [233]. A small number of studies, including gefitinib, have utilized EGFR receptor saturation as part of an endpoint in phase 1 studies [234–236]. However, it is unclear if adequate EGFR receptor saturation is correlated to efficacy and toxicity and receptor turnover as a result of EGFR TKIs binding, as there is a potential limitation in measuring receptor saturation due to the short half-life of about 12 h [237, 238].

Immunotherapy has been explored as an alternative or complementary therapeutic strategy among lung cancer patients, particularly in those with TKIs resistance and/or in advanced stages of EGFR mutant lung cancers and are unaffected by the genetic polymorphisms of drug metabolizing enzymes and/or transporters [239, 240]. EGFR-directed monoclonal antibodies such as cetuximab, necitumumab, panitumumab, matuzumab and nimotuzumab can bind on to EGFR present on the surface of tumor cells and prevent the binding of the ligand epidermal growth factor (EGF) in the extracellular domain, resulting in inhibition of EGFR signalling. These monoclonal antibodies could also inhibit EGFR signaling by other mechanisms including antibody dependent cellular toxicity (ADCC) [241]. However, lack of significant clinical benefits with the combination therapy of EGFR-directed monoclonal antibodies with TKIs in EGFR mutant lung cancer patients warrants the need for further evidence [242]. Immune check point inhibitors (ICIs), comprising of monoclonal antibodies against programmed cell death protein-1 (PD-1) such as nivolumab, pembrolizumab and programmed death-ligand1 (PD-L1) such as atezolimumab and durvalumab, have been reported to improve the clinical response in few subsets of lung cancer patients. However, majority of the available reports suggest that EGFR-mutant lung cancer patients have shown poorer response to ICIs treatment [243–246]. Several factors such as lower PD-L1 expression and tumor mutational burden, increased risk of pulmonary toxicity in patients on prior or concurrent osimertinib therapy, limited efficacy with ICIs monotherapy and risk of developing hyper-progressive disease (HPD), warrant caution for their use in EGFR mutant lung cancer patients [246, 247].

## Challenges of liquid biopsy-based detection and PGx of EGFR mutation and resistant lung cancer

**Fig. 4:**
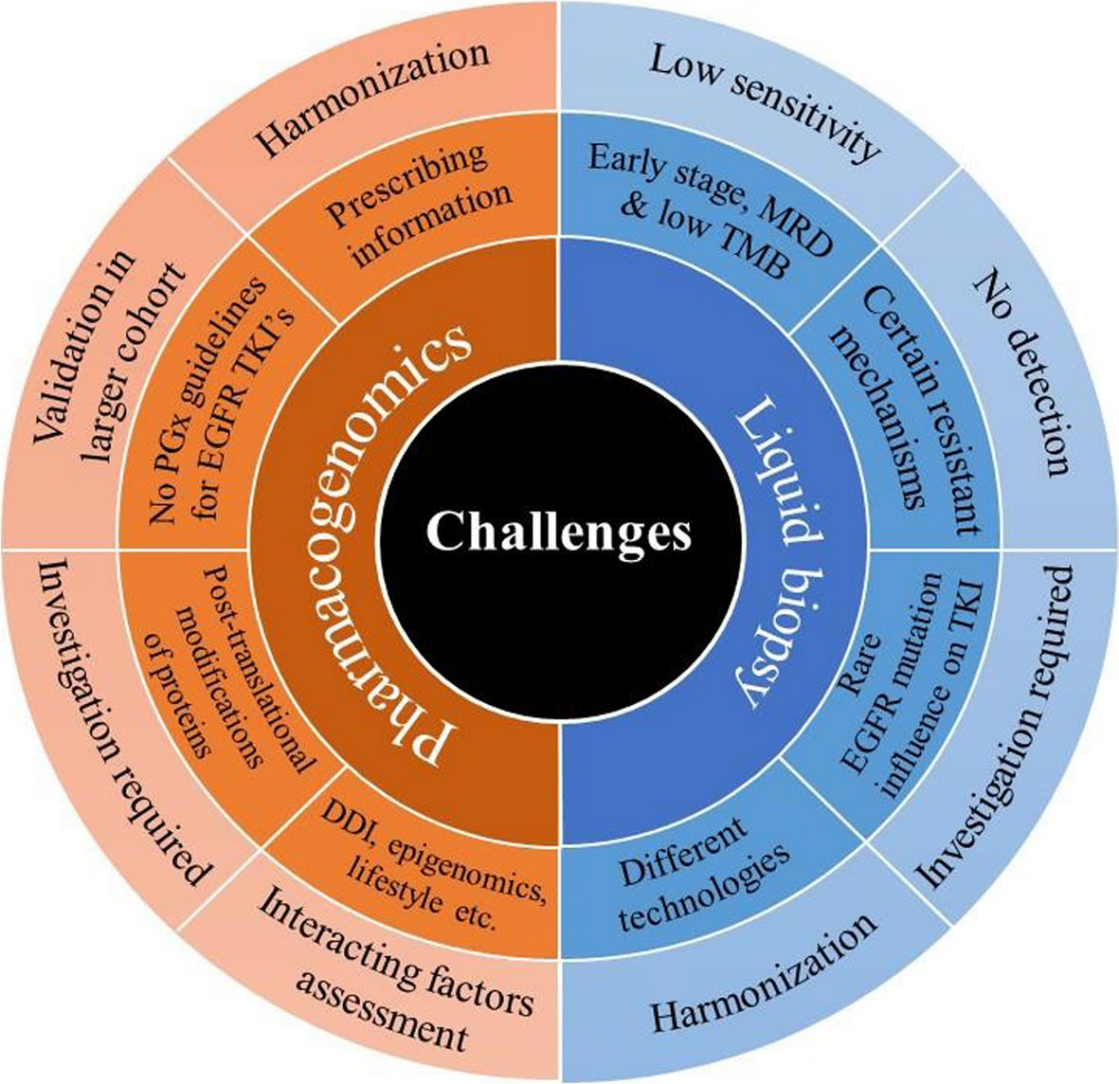
Challenges faced by liquid biopsy and PGx for their implementation in clinical practice for EGFR resistant and mutant lung cancer patients. Abbreviations: PGx: Pharmacogenomics; EGFR: Epidermal growth factor receptor; TKIs: Tyrosine kinase inhibitors; PTM: Post translational modifications; DDI: Drug-drug interaction; MRD: Minimal residual disease; TMB: Tumor mutational burden

EGFR T790M mutation accounts for only about half of the resistance mechanisms in NSCLC patients who developed acquired resistance to first or second-generation TKIs. Liquid biopsy may not be able to detect other resistance mechanisms, such as small cell cancer transformation [80]. Several rare EGFR mutations are known to cause conformational alterations in the EGFR drug binding region. However, their influence on TKIs responses is still debated and require further clinical validation [248, 249]. Some liquid biopsy assays have been reported to have a lower sensitivity for EGFR mutations compared to tissue biopsy that may be attributed to sampling from different tumor cell populations as well as differing sequencing technologies [137, 250]. An increased frequency of EGFR T790M detection correlated with tumor progression/ metastasis by liquid biopsy and is explained by low copy number in peripheral blood in early stage, that may pose problems for early screening of lung cancer by liquid biopsy [138, 251]. Further, the TMB in EGFR-mutated tumors was shown to be significantly lower than in EGFR wild-type tumors [252]. Robust implementation of liquid biopsy as a clinical tool in the management of EGFR resistant lung cancer warrants further harmonization of the diverse ctDNA analysis technologies and different platforms, and requires multicentric randomized controlled trials with larger cohorts of patients and controls [253]. Harmonization of PGx guidelines among different consortia and agencies and lack of compliance among physicians for PGx label-based testing and prescribing present key challenges in the implementation of pharmacogenomics-based therapy management in clinics [254]. PGx does not offer information on the post-translational modifications of encoded proteins, therefore the importance of this element in cancer therapy requires additional investigations [255]. Implications of other interacting factors on genetic polymorphisms of CYP450 enzymes and drug transporters such as various patient specific factors, ethnicity, epigenomics, lifestyle, drug-drug and drug-dietary interactions could pose challenges in deriving appropriate genotyping-based dosage implementation at an individual level in clinics [255–257]. Most importantly, currently there is a lack of guidelines supporting testing of CYP450 and drug transporter polymorphisms to guide dosing of EGFR TKIs. A summary of these limitations is represented in Fig. 4.

## Conclusion

While tissue remains the most accepted material for molecular profiling of solid tumors such as lung cancers, it is limited by the dynamic and heterogenous nature of cancer resulting in spontaneous occurrence of clonal evolution and drug resistance. Liquid biopsy has emerged as an imperative alternative and/or complementary tool to tissue biopsy for molecular profiling in lung cancer due to its relative advantages such as being minimally invasive, reduced procedural complications, as well as its utility for longitudinal monitoring of patients for monitoring acquired resistance to TKIs. An integrated approach of employing liquid biopsy and PGx for serial molecular profiling of EGFR mutant and resistant lung cancer patients at an individual level as well as at population subsets could represent a potential precise screening and monitoring tool in this era of precision oncology by identifying precise doses of TKIs against targetable EGFR mutations. Though significant progress has been made in these fields, several aspects related to their successful implementation in practice, such as framing of robust guidelines, harmonization of sequencing technologies and platforms, multicentric validation in larger patient cohorts, and identification of various interacting factors needs to be addressed before clinical adoption at a global scale.

## Supplementary Information

**Additional file 1: Supplementary Table 1.** Completed and ongoing trials on liquid biopsy for the detection of EGFR mutant NSCLC.

## Data Availability

“Not applicable”.

## Abbreviations

ABC: Adenosine triphosphate binding cassette
ABCB1: Adenosine triphosphate binding cassette subfamily B member 1
ABCG2: Adenosine triphosphate binding cassette subfamily G member 2
ADCC: Antibody dependent cellular toxicity
AKT: Ak strain transforming murine thymoma viral oncogene homolog
ALK: Anaplastic lymphoma kinase
ASCO: American Society of Clinical Oncology
ATP: Adenosine triphosphate
AUC: Area under the curve
AXL: Anexelekto
BCRP: Breast cancer resistant protein
BLACAT1: Bladder cancer associated transcript 1
BRAF: Rapidly accelerated fibrosarcoma viral oncogene homolog B1
BRAFV600E: B-Raf proto-oncogene; valine (V) is substituted by glutamic acid (E)
bTMB: Blood tumor mutational burden
CAPP-Seq: Cancer personalized profiling by deep sequencing
ccfDNA: Circulating cell free DNA
CDK6 CNG: Cyclin-dependent kinase 6 copy number gain
CDK6: Cyclin-dependent kinase 6
CDKN2A: Cyclin-dependent kinase inhibitor 2A
cfDNA: Cell free DNA
circRNA: Circular RNA
Cmax: Maximum serum concentration
c-MET: Mesenchymal epithelial transition factor
CNS: Central nervous system
CTCs: Circulating tumor cells
ctDNA: Circulating tumor DNA
CTNNB1: Catenin beta1
ctRNA: Circulating tumor RNA
CYP450: Cytochrome P450
DDI: Drug drug interaction
ddPCR: Digital droplet polymerase chain reaction
DNA: Deoxyribonucleic acid
dPCR: Digital polymerase chain reaction
E-Cadherin: Epithelial cadherin
EDTA: Ethylenediamine tetraacetic acid
EFIRM: Electric field-induced release and measurement
EGF: Epidermal growth factor
EGFR: Epidermal growth factor receptor
EM: Extensive metabolizers
EpCAM: Epithelial cell adhesion molecule
ErbB: Erythroblastic leukemia viral oncogene homologue
ERBB2: Erythroblastic leukemia viral oncogene homolog 2
ERBB3: Erythroblastic leukemia viral oncogene homolog 3
ERK: Extracellular signal regulated kinase
ESMO: European Society for Medical Oncology
EVs: Extracellular vesicles
ExoNA: Exosomal RNA/DNA and cfDNA
ExoTNA: Exosomal DNA and RNA
FDA: Food and Drug Administration
HER2: Human epidermal growth factor receptor 2
HGF: Hepatocyte growth factor
HPD: Hyper-progressive disease
ICIs: Immune check point inhibitors
IM: Intermediate metabolizers
JAK: Janus activated kinase
KRAS: Kirsten rat sarcoma
lncRNA: Long non-coding RNA
MALAT-1: Metastasis associated lung adenocarcinoma transcript 1
MAPK: Mitogen-activated protein kinase
MDRPs: Multi-drug resistant transporter proteins
MEK: Mitogen-activated protein kinase kinase
MET: Mesenchymal epithelial transition factor
miRNA: MicroRNA
MRD: Minimal residual disease
mRNA: Messenger RNA
mTOR: Mammalian target of rapamycin
NCCN: National Comprehensive Cancer Network
NGS: Next generation sequencing
NM: Normal metabolizers
NR1 I3: Nuclear receptor subfamily 1 group I member 3
NSCLC: Non-small cell lung cancer
NTRK: Neurotrophic tropomyosin receptor kinase
ORR: Overall response rate
OS: Overall survival
PAH: Polycyclic aromatic hydrocarbons
PD: Pharmacodynamic
PD-1: Programmed cell death protein-1
PD-L1: Programmed death-ligand 1
PFS: Progression-free survival
P-gp: Permeability glycoprotein
PGx: Pharmacogenomic
PIK3CA: Phosphatidylinositol 4,5-bisphosphate 3-kinase catalytic subunit alpha isoform
PK: Pharmacokinetic
PM: Poor metabolizers
PTMs: Post translational modifications
qPCR: Quantitative polymerase chain reaction
RAF: Rapidly accelerated fibrosarcoma
RAS: Rat sarcoma virus
RB: Retinoblastoma
RCTs: Randomized controlled trials
RECIST: Response Evaluation Criteria in Solid Tumors
RET: Rearranged during transfection
RNA: Ribonucleic acid
ROS1: Receptor tyrosine kinase
RT-qPCR: Quantitative real-time polymerase chain reaction
SCLC: Small-cell lung cancer
SNPs: Single nucleotide polymorphisms
STAT3: Signal transducer and activator of transcription 3
TAT: Turnaround time
TDM: Therapeutic drug monitoring
TEPs: Tumor educated platelets
TKIs: Tyrosine kinase inhibitors
TMB: Tumor mutational burden
TRACERx: Tracking non–small-cell lung cancer evolution through therapy
tTMB: Tissue tumor mutational burden
UM: Ultra rapid metabolizers
WBC: White blood cell
WES: Whole exome sequencing
WHO: World Health Organization
WNT: Wingless-related integration site
ZEB1: Zinc finger E-box-binding homeobox 1
