# Supplementary material for: Integration of liquid biopsy and pharmacogenomics for precision therapy of EGFR mutant and resistant lung cancers
Source: Mol Cancer. 2022 Feb 24;21:61. doi: 10.1186/s12943-022-01534-8 (PMC8867675; doi:10.1186/s12943-022-01534-8)
Supplement: Supplementary file 1 — Additional file 1: Supplementary Table 1. Completed and ongoing trials on liquid biopsy for the detection of EGFR mutant NSCLC. [file 12943_2022_1534_MOESM1_ESM.docx]

Supplementary Table 1: Completed and ongoing trials on liquid biopsy for the detection of EGFR mutant NSCLC.

| **S. No** | **NCT** | **Sample size** | **Mutation directed** | **EGFR mutation** | **Study type** | **Status** | **Outcome** |
| --- | --- | --- | --- | --- | --- | --- | --- |
| 1 | NCT02906852 | 264 | EGFR, ALK |  | Observational | **Completed**  December, 2018 | - Concordance in the detection of molecular abnormalities using Inivata's liquid biopsy panel with detection using standard of care tissue biopsy analysis. - Proportion of NSCLC patients eligible for targeted therapy based on liquid biopsy analysis as an evaluation of feasibility molecular stratification, compared to standard of care alone. - Detection: sensitivity and specificity of molecular abnormalities using Inivata liquid biopsy panel relative to standard of care tissue biopsy analysis. |
| 2 | NCT02771314 | 48 | EGFR | T790M, C797S, L858R, del 19 EGFR mutations | Interventional | **Unknown**  December, 2020 | - Biomarkers of resistance to first and third (AZD9291) generation EGFR TKIs, explored by serum or plasma DNA specimens and baseline CTCs. |
| 3 | NCT03059641 | 300 | EGFR, ALK, ROS1, MET exon 14 skipping mutation |  | Observational | **Completed**  January, 2020 | - The mutation pattern of untreated advanced NSCLC and evolution of ctDNA mutation profile during TKI treatment. - The concordance of gene mutation pattern between liquid biopsy and tissue biopsy. |
| 4 | NCT02980536 | 129 | EGFR | Activating | Observational | **Unknown**  December, 2018 | - The TKI resistance gene mutation pattern in plasma DNA of TKI treated patient. - The concordance of gene mutation pattern between liquid biopsy and traditional cancer tissue biopsy. |
| 5 | NCT03228277 | 25 | EGFR | G719X, exon 19 deletion, L858R, L861Q | Interventional | **Completed**  July, 2019 | - Objective response rate. |
| 6 | NCT03615443 | 306 | EGFR, ALK, ROS1, BRAF, MET |  | Observational | **Unknown**  November, 2020 | - Demonstrate the non-inferiority of cfDNA-based vs. tumor tissue-based genotyping. - Turnaround time for cfDNA vs. tissue results. - Tumor not detected rate of cfDNA in blood. |
| 7 | NCT04703153 | 200 | EGFR, ALK, RET, ROS1, NTRK fusions, MET, BRAF, ERBB2 and KRAS |  | Observational | **Ongoing**  November, 2023 | - Demonstration of the non-inferiority of cfDNA-based liquid biopsy assay vs. tissue biopsy based NGS assay for mutation profile results. |
| 8 | NCT04912687 | 580 | EGFR |  | Interventional | **Ongoing**  October, 2023 | - To assess the detection rate of patients with an EGFR actionable alteration when using the combination of two diagnostic procedures which include liquid biopsy analysis (by droplet digital PCR or allele specific PCR) and tissue analysis. - The detection rate of patients with an EGFR actionable alteration based on the use of liquid biopsy analysis only. |
| 9 | NCT03771404 | 50 | AKT1, KRAS, NRAS, BRAF, DDR2, EGFR, FGFR1, ERBB2 (HER2), MEK1, MET, PIK3CA, PTEN, TP53, MDM2, SOX2 and P63. |  | Interventional | **Ongoing**  December, 2021 | - To evaluate whether the individual patient's molecular landscape of ctDNA and CTCs could be reliable biomarkers for the early prediction of disease relapse. - To monitor using "liquid biopsy" the tumor clonal evolution during the post operation period and define a correlation between the genotype of the primary tumor and the emergence of molecularly different clones. - To investigate the potential of longitudinal "liquid biopsy" to predict the genetic profile of metastasis. |
| 10 | NCT02511288 | 900 | EGFR, BRAF, HER2, ALK, ROS1, |  | Observational | **Ongoing**  December, 2026 | - Identification of the genetic profile in advanced or metastatic NSCLC patients using liquid biopsies (circulating tumoral DNA). - Evaluation of the liquid biopsy role in the tumoral monitoring. - Circulating tumoral cells isolation and analysis to determine the role of non-genomic and/or phenotypic factors in the treatment’s response. |
| 11 | NCT03865511 | 66 | EGFRm+, HER2, cMET expression and/or amplification | Ex19 deletions, L858R, C797S | Interventional | **Ongoing**  July, 2024 | - Examination of the genetic profile at the point of disease progression in EGFRm+ patients receiving osimertinib as first-line EGFR TKI therapy compared to baseline. |
| 12 | NCT02759835 | 37 | EGFR | EGFR-sensitizing somatic mutations or a germline T790M mutation | Interventional | **Ongoing**  September, 2022 | - Examination of the genetic profile at the point of disease progression in EGFRm+ patients receiving osimertinib as first-line EGFR TKI therapy compared to baseline. - To demonstrate that the early kinetics of ctDNA is an indicator of response to osimertinib. - To compare the genetic profile of the ctDNA and the tumor biopsy. |
| 13 | NCT04372732 | 200 | EGFR (-); ALK (-), ROS1 (-). |  | Observational | **Ongoing**  August, 2022 | - The correlations of tumor autoantibodies and PFS/ORR of PD-1 blockade treatment. |
| 14 | NCT02633189 | 200 | EGFR | Exon19 deletion or exon 21 L858R mutation or activating/sensitizing mutations, such as exon 21 L861Q, exon 18 G719S, G719A and G719C, exon 20 S768I and V769L  Excluding T790M alone | Interventional | **Ongoing**  July, 2022 | - Progression free survival according to type of EGFR mutation (exon 19del, exon 21L858R, other). - Number and type of EGFR mutations in plasma samples. |
| 15 | NCT05020275 | 60 | EGFR | Deletion of exon 19 or L858R, L861x, or G719x mutation | Observational | **Ongoing**  March, 2023 | - Relationship between plasma exposure to osimertinib and response to treatment assessed by PFS. - Longitudinal correlation between the plasma concentration of osimertinib and the concentration of ctDNA (liquid biopsy). - Correlation between the trough plasma concentration of osimertinib and the time to onset of acquired molecular resistance mutations to osimertinib (identified on ctDNA). - Correlation between the concentration of ctDNA and acquired clinical resistance - Influence of genetic polymorphisms on the plasma concentration of osimertinib (CYP3A4 and ABCB1). - The intra-individual variability of osimertinib plasma concentration. |
| 16 | NCT04564079 | 200 | EGFR, ROS1, ERBB2, MET, BRAF, KRAS, RET, ALK |  | Observational | **Ongoing**  August, 2023 | - Clinical utility of returning genomic aberration results by quantifying the number of patients who received targeted therapy based on results returned from the Oncomine Precision Assay genomic aberration results in blood and/or tissue. - Clinical utility of returning genomic aberration results by measuring the turnaround time (in days) from the date of request for SOC genetic testing in tissue versus date of blood collection for the Oncomine Precision Assay to the report date. |
| 17 | NCT04285671 | 26 | EGFR | Activating and sensitizing  [literature support for mutations other than exon 19 deletion and the L858R point mutation] | Interventional | **Ongoing**  December, 2023 | - Identification of the recommended phase II dose (R2PD) regimen for combination osimertinib, necitumumab, trastuzumab (ONT) therapy (Phase Ib). - Potential biomarkers associated with response from liquid biopsies. - Mutant allele fraction in ctDNA. |

Abbreviations: EGFR: Epidermal growth factor receptor; NSCLC: Non-small cell lung cancer; ALK: Anaplastic lymphoma kinase; TKIs: Tyrosine Kinase Inhibitors; CTCs: Circulating tumor cells; ROS1: Receptor tyrosine kinase; MET: Mesenchymal epithelial transition factor; ctDNA: Circulating tumor DNA; BRAF: Rapidly accelerated fibrosarcoma viral oncogene homolog B1; cfDNA: Cell free DNA; RET: Rearranged during transfection; NTRK: Neurotrophic tropomyosin receptor kinase; ERBB2: Erythroblastic leukemia viral oncogene homolog 2; KRAS: Kirsten rat sarcoma; NGS: Next generation sequencing; PCR: Polymerase Chain Reaction; AKT1: Ak strain transforming murine thymoma viral oncogene homolog 1; NRAS: Neuroblastoma rat sarcoma viral oncogene homolog; DDR2: Discoidin domain receptor 2; FGFR1: Fibroblast growth factor receptor 1; MEK1: Mitogen- activated protein kinase kinases 1; PIK3CA: Phosphatidylinositol 4,5-bisphosphate 3-kinase catalytic subunit alpha isoform; PTEN: Phosphatase and tensin homolog; TP53: Tumor protein p53; MDM2: Mouse double minute 2 protein; SOX2: Sry-related HMG box 2; p63: Tumor protein 63; EGFRm+: mutated Epidermal Growth Factor Receptor; HER2: Human epidermal growth factor receptor 2; PFS: Progression-free survival; ORR: Overall response rate; Programmed cell death protein 1; ABCB1: ATP binding cassette subfamily B member 1; SOC: Standard of care; R2PD: Recommended phase II dose; DNA: Deoxyribonucleic acid.
